# Outcomes of general paediatric surgical neonates managed at the Nelson Mandela Children’s Hospital, Johannesburg, South Africa

**DOI:** 10.1007/s00383-026-06365-y

**Published:** 2026-02-27

**Authors:** Sphamandla Zulu, Karen Milford, Andrew Grieve

**Affiliations:** 1 Department of Paediatric Surgery, Nelson Mandela Children’s Hospital, Johannesburg, South Africa; 2 Department of Paediatric Surgery, University of the Witwatersrand, Johannesburg, South Africa

**Keywords:** Neonates, Paediatric surgery, SNAPPE II, Risk stratification, Sepsis, NICU

## Abstract

**Background:**

Early identification of high-risk surgical neonates is essential in resource-limited settings. Risk stratification tools such as the Score for Neonatal Acute Physiology with Perinatal Extension II (SNAPPE II) are widely used in neonatal intensive care units but have not been validated in surgical neonatal populations in sub-Saharan Africa. This study evaluated outcomes and the prognostic utility of SNAPPE II in surgical neonates.

**Methods:**

A 5-year retrospective cohort study was conducted including neonates admitted under general paediatric surgery to the neonatal intensive care unit at Nelson Mandela Children’s Hospital. SNAPPE II scores were calculated within 12 h of admission. Associations between SNAPPE II and mortality were assessed using relative risk, odds ratios, and receiver operating characteristic (ROC) analysis.

**Results:**

380 neonates were analysed. Overall mortality was 22.6%, with sepsis accounting for 65.1% deaths. SNAPPE II demonstrated strong prognostic performance: scores > 20 were associated with markedly increased mortality (relative risk 9.2, odds ratio 43.7), and no neonates survived with scores ≥ 40. ROC analysis showed excellent discrimination (AUC 0.88), with an optimal cut-off of 25.

**Conclusion:**

SNAPPE II reliably predicts mortality in surgical neonates and supports early triage, rational resource allocation, and informed family counselling in resource-limited neonatal surgical units.

**Supplementary Information:**

The online version contains supplementary material available at 10.1007/s00383-026-06365-y.

## Introduction

The Nelson Mandela Children’s Hospital (NMCH) is a tertiary and quaternary level, specialist paediatric referral hospital located in Johannesburg, South Africa. It hosts the largest critical care complex in the Southern African Development Community (SADC), offering a total of 48 neonatal and paediatric intensive care unit (ICU) beds. General paediatric surgical services commenced in July 2018. NMCH does not have a maternity unit and receives referrals from across South Africa and neighbouring countries.

The neonatal period is the most vulnerable stage of life, with the highest risk of mortality. In 2019, the global average neonatal mortality rate was 18 per 1,000 live births, and most under-five deaths occurred during this phase [1]. Globally, the leading causes of neonatal deaths include birth asphyxia, infections, and prematurity [2]. Outcomes for neonatal surgery differ markedly between high-income countries (HICs) and low- and middle-income countries (LMICs), with mortality in HICs declining from 50.0% in the 1950s to under 5.0% today [3, 4]. In contrast, African nations report surgical neonatal mortality rates ranging from 16.0 to 45.0% [4]. Understanding these disparities is crucial, especially in middle-income countries like South Africa, where tertiary level paediatric care is evolving.

Congenital anomalies contribute to approximately 10.0% of neonatal deaths in Sub-Saharan Africa (SSA), many of which require surgical intervention [1, 5, 6]. These anomalies account for over half of emergency paediatric operations and a quarter of surgical admissions in African settings [5]. Despite being a middle-income country, South Africa has mortality rates comparable to other SSA nations [6, 7].

Improved outcomes rely on access to NICU-level care and specialised surgical services [8–10]. Most hospitals in SSA lack these resources, highlighting the need for regional referral centres. Sepsis remains a major cause of postoperative morbidity and mortality in neonates [6, 7, 11, 12].

To enhance prognostication in this high risk group, scoring systems such as the Score for Neonatal Acute Physiology with Perinatal Extension II (SNAPPE II), though validated for NICUs broadly, remain underutilized in surgical neonates. The aim of this study was to evaluate factors associated with outcomes in surgical neonates, with particular focus on the impact of physiological illness severity, as measured by the SNAPPE II score, on mortality.

## Methods

A retrospective cohort study was conducted, evaluating all neonates admitted under the general paediatric surgery service to the neonatal intensive care unit (NICU) at the Nelson Mandela Children’s Hospital (NMCH) over a five-year period, from 1 July 2018 to 30 June 2023. Ethical approval was obtained from the University of the Witwatersrand Human Research Ethics Committee (Approval number: M230981) and institutional approval from Nelson Mandela Children’s Hospital.

Data were retrieved from multiple sources, including the NMCH medical records, operative notes, NICU admission registers, theatre logbooks, and the institutional database maintained by the Department of Paediatric Surgery at the University of the Witwatersrand.

Inclusion criteria comprised all neonates admitted to the NICU under general paediatric surgery during the five year study period. Exclusion criteria included neonates not managed by general paediatric surgery and those with significantly incomplete clinical or operative records.

Data were collected and managed using a password-protected Microsoft Excel spreadsheet. Statistical analysis was conducted using Stata software, version 17. Descriptive statistics, including measures of central tendency (mean, median), frequencies, and percentages, were used to summarize demographic and clinical variables.

Inferential statistics were used to evaluate associations between demographic variables and clinical outcomes. Chi-square tests and Fisher’s exact test (applied when expected cell counts were < 5) were used for categorical comparisons. Normality of continuous variables was assessed using distribution plots and correlation analysis. Skewness and kurtosis were evaluated to determine data symmetry and shape. Parametric tests were applied where assumptions of normality were satisfied; otherwise, non-parametric alternatives were used.

Parametric comparisons of continuous variables were performed using Student’s t-test where assumptions of normality were met, while non-parametric comparisons were conducted using the Mann–Whitney *U* test when data were not normally distributed. All categorical variables are reported as absolute numbers with denominators (n/N) alongside corresponding percentages.

Univariable analyses were performed to evaluate associations between individual demographic and clinical variables and outcomes. Given that SNAPPE II is a composite severity score incorporating multiple physiological parameters, multivariable models including SNAPPE II were not constructed to avoid collinearity and over-adjustment. The analytical approach was therefore descriptive and prognostic rather than causal.

A p-value of < 0.05 was considered statistically significant. Where appropriate, 95% confidence intervals were reported to indicate the precision of effect estimates.

## Results

### Study population

A total of 422 neonates were eligible for inclusion, 42 were excluded because of incomplete clinical and operative records, leaving 380 neonates for analysis. Of these, 377 underwent surgical intervention. Three neonates died before surgery, two with congenital diaphragmatic hernia (CDH) and one with necrotizing enterocolitis (NEC).

### Referral patterns

Table 1 outlines the birth characteristics and referral details. A majority were born preterm 260/380 (68.0%), with the largest subgroup comprising moderate to late preterm neonates. Low birth weight was common, affecting 239/380 (62.8%) of the cohort when combining extreme, very low, and low birth weight categories. Human immunodeficiency virus (HIV) exposure was documented in 69/380 (18.0%) of neonates, all testing negative on birth polymerase chain reaction (PCR) and all received antiretroviral prophylaxis.

**Table 1:** Provides demographic and clinical characteristics for the cohort of 380 neonates

Characteristic	Value
*Demographics*	
Age (days)	4.0 (IQR 2.0, 8.0)
*Gender*	
Female	171 (45%)
Male	209 (55%)
*Birth weight*	
Extreme low birth weight	7 (1.8%)
Very low birth weight	50 (13%)
Low birth weight	182 (48%)
Normal birth weight	141 (37%)
*Birth and delivery details*	
Gestation (weeks)	36.00 (IQR 33.00, 38.00)
Gestation	
Extremely preterm	7 (1.8%)
Moderate to late preterm	204 (54%)
Term	120 (32%)
Very Preterm	49 (13%)
*Mode of delivery*	
Caesarean section	112 (29%)
Normal vaginal delivery	268 (71%)
*Place of delivery*	
Home	2 (0.5%)
Hospital	378 (99%)
***Diagnosis***	
*Antenatal diagnosis*	
None with polyhydramnios	8 (2.1%)
None without polyhydramnios	371 (98%)
Posterior urethral valves	1 (0.3%)
*HIV Exposure*	
No	311 (82%)
Yes	69 (18%)
*ARV prophylaxis*	
No prophylaxis	297 (78%)
Not required	13 (3.4%)
Prophylaxis given	70 (18%)
*Birth PCR result*	
Negative PCR	70 (18%)
Not required	310 (82%)
***Referring institution***	
*Distance of referral hospital*	
20 km or less	90 (24%)
20–100 km	172 (45%)
101–200 km	41 (11%)
201–300 km	28 (7.1%)
301–400 km	38 (10%)
Above 400 km	11 (2.9%)

A total of 262/380 (69.0%) referrals to NMCH originated from healthcare facilities within a 100 km radius, while 118/380 (31.0%) were referred from distances exceeding 100 km. Supplementary Fig. 1 illustrates the geographic distribution of referral institutions to our centre.

### SNAPPE II scoring

SNAPPE II scores were calculated using clinical and physiological parameters recorded within the first 12 h of NICU admission (Table 2). Most neonates 256/380 (67.3%) scored between 0 to 9 and had significantly better outcomes with an 82.0% survival rate (p value < 0.001). A statistically significant linear trend was observed between increasing SNAPPE II scores and mortality (p < 0.001, Chi-square test for trend). No neonates had scores ≥ 80.

**Table 2:** SNAPPE II scores of 380 neonates, stratified by score into standard 10-point intervals, ranging from 0–9 up to ≥ 80

SNAPPE II Score Category	Overall N = 380	Non-survivors N = 86	95% CI	Survivors N = 294	95% CI
0–9	256.00 (67.3%)	15.00 (17.4%)	10%, 27%	241.00 (81.9%)	77%, 86%
10–19	51.00 (13.4%)	12.00 (13.9%)	7.7%, 23%	39.00 (13.2%)	9.7%, 18%
20–29	20.00 (5.2%)	14.00 (16.2%)	9.5%, 26%	6.00 (2.0%)	0.83%, 4.6%
30–39	33.00 (8.6%)	25.00 (29.0%)	20%, 40%	8.00 (2.7%)	1.3%, 5.5%
40–49	15.00 (3.9%)	15.00 (17.4%)	10%, 27%	0.00 (0.0%)	0.00%, 1.6%
50–59	2.00 (0.5%)	2.00 (2.3%)	0.40%, 8.9%	0.00 (0.0%)	0.00%, 1.6%
60–69	2.00 (0.5%)	2.00 (2.3%)	0.40%, 8.9%	0.00 (0.0%)	0.00%, 1.6%
70–79	1.00 (0.2%)	1.00 (1.1%)	0.06%, 7.2%	0.00 (0.0%)	0.00%, 1.6%
> = 80	0.00 (0.0%)	0.00 (0.0%)	0.00%, 5.3%	0.00 (0.0%)	0.00%, 1.6%

Outcomes were grouped into two categories, non-survivors and survivors. Proportions and 95% confidence intervals (CI) were calculated for each score range within both groups. Fisher’s exact test was used to assess the association between SNAPPE II categories and mortality

A threshold effect was evident, scores > 20 were associated with markedly increased mortality. Neonates with scores > 20 had a ninefold higher risk of death compared to those with scores < 20 (59/73 [80.8%] vs 27/307 [8.8%]), corresponding to a relative risk of 9.2 (95% CI 6.3–13.4) and an odds ratio of 43.7 (95% CI 21.6–88.4).

Supplementary Fig. 2 illustrates that survival was concentrated in the lowest SNAPPE II category (0–9), with a stepwise decline as scores increased and no survivors with scores above 40, demonstrating the score’s strong discriminatory capacity and its clinical value for early risk stratification in surgical neonates.

### Morbidity profile

Postoperative complications were classified using the Clavien-Dindo system. The most frequent complication was Grade 3b (n = 37), requiring surgical re-intervention under general anaesthesia (Supplementary Fig. 3), followed by Grade 1 (n = 22), which involved minor interventions. Severe complications were rare: one case each of Grade 4a, 4b, and 5. No Grade 3a events occurred.

Surgical site infection (SSI) was the most common specific complication (36.6% of all morbidities), followed by anastomotic leak (n = 18) and anastomotic stricture (n = 12). Less frequent complications included enterocutaneous fistula, iatrogenic bowel injury, and sheath dehiscence (supplementary Fig. 4).

### Mortality analysis

The overall mortality rate was 86/380 (22.6%). Of these, 70/380 (18.4%) of neonates died within 30 days of admission, while 16/380 (4.2%) died thereafter. Early neonatal mortality (death within the first 7 days of life) occurred in 15/380 (3.9%) of cases.

Sepsis was the leading cause of death, accounting for 56/86 (65.1%) of mortalities. Palliation, defined as the withdrawal of care due to a poor prognosis or congenital anomalies incompatible with life, contributed to 12/86 (13.9%) of deaths. Additional causes included cardiac failure 10/86 (11.6%), multi-organ failure 7/86 (8.1%) and pulmonary haemorrhage 1/86 (1.1%). All sepsis-related deaths in this cohort were culture-confirmed, with no cases classified as clinical sepsis without microbiological confirmation.

The most frequently isolated pathogen in fatal sepsis cases was Acinetobacter baumannii (32.1%), followed by polymicrobial infections (28.3%), and Klebsiella pneumoniae (20.8%). Less frequent organisms included Staphylococcus epidermidis (9.4%), methicillin-resistant Staphylococcus aureus (MRSA) (5.7%), Enterococcus faecalis, and Serratia marcescens.

Palliation was primary because of bowel length < 15 cm (25.0%), midgut necrosis (16.7%) and NEC totalis (16.7%). Other reasons included complex cardiac lesions, severe intraventricular haemorrhage, multiple congenital anomalies, and Trisomy 18.

Among the 86 neonatal deaths, NEC (n = 22 cases; 25.6%) and gastroschisis (n = 20; 23.3%) were most frequent. Complex gastroschisis had a significantly higher mortality (73.3%) compared to simple (14.5%) (p < 0.001). Intestinal atresia and oesophageal atresia each contributed to 11 deaths (12.8%). Anorectal malformations (ARM) and congenital diaphragmatic hernia (CDH) accounted for 6 deaths each (7.0%). Notably, all ARM-associated deaths occurred in neonates with concomitant complex congenital cardiac anomalies. Other fatal diagnoses included gastric perforation (n = 4), intestinal malrotation (n = 2), and miscellaneous causes (n = 4).

### Positive blood cultures and mortality

**Fig. 1:**
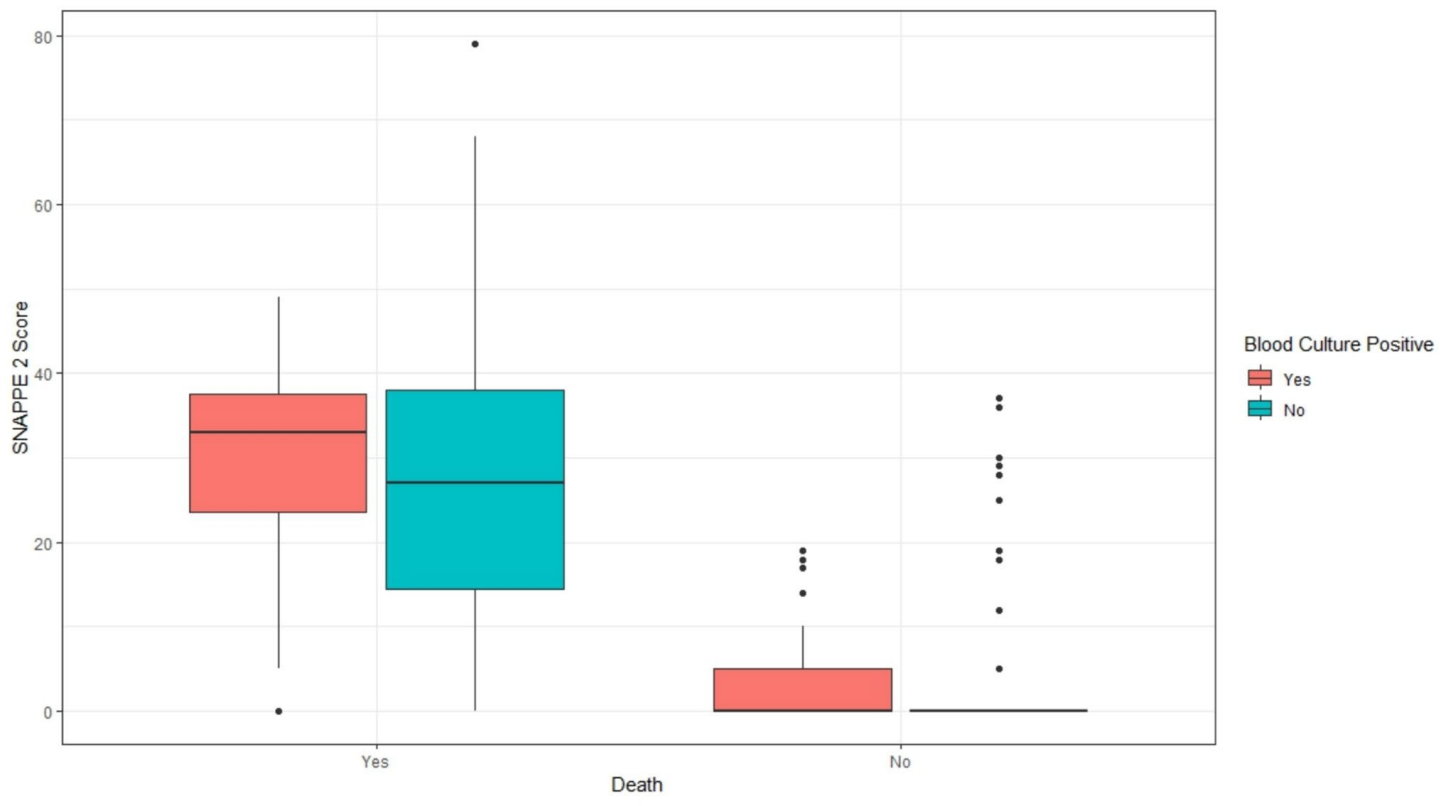
Box-and-whisker plot illustrating the distribution of SNAPPE II scores stratified by mortality outcome (death vs. survival) and blood culture status (positive vs. negative). Patients were grouped into four subcategories based on survival and culture results. The plot displays the median, interquartile range (IQR), and outliers for each subgroup. This visualization highlights the association between illness severity, culture-positive sepsis, and clinical outcome. Statistical comparisons between groups were conducted using non-parametric tests, as appropriate

Of the 52 neonates with positive blood cultures, 27 (51.9%) died, compared to 59 deaths among the 328 with negative cultures (18%), yielding an odds ratio of 4.92 (95% CI: 2.67–9.09; p < 0.001). This underscores the prognostic significance of culture-positive sepsis and supports aggressive early intervention. Figure 1 shows that median SNAPPE II scores were higher among non-survivors, particularly those with positive blood cultures. Survivors had low, tightly clustered scores regardless of culture status.

### Survival and risk stratification

**Fig. 2:**
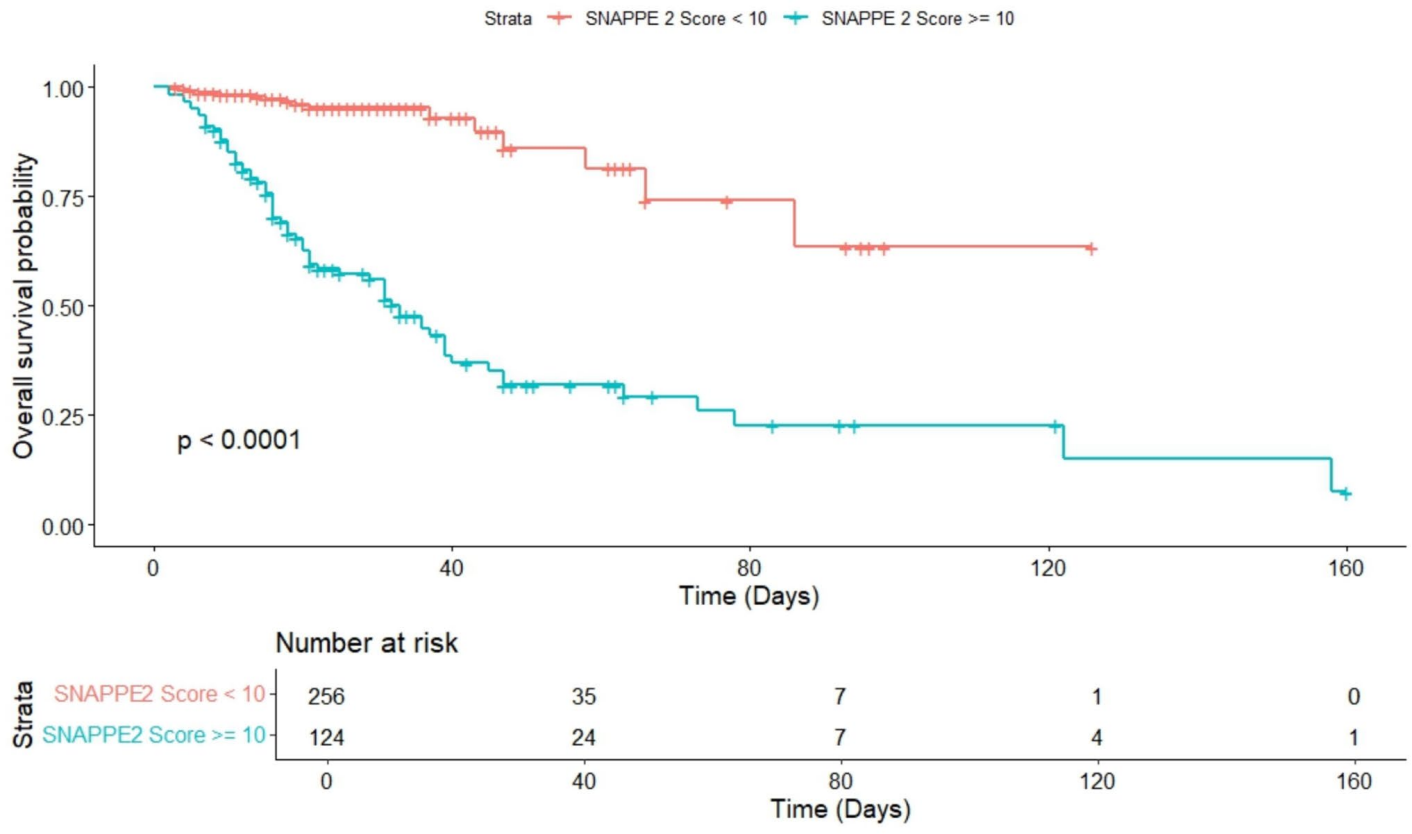
A Kaplan–Meier survival analysis was conducted to evaluate the relationship between SNAPPE-II scores and overall survival probability among neonates admitted for surgical care. Patients were stratified into two groups based on SNAPPE-II score: SNAPPE-II < 10 (lower risk group) and SNAPPE-II ≥ 10 (higher risk group)

For descriptive survival analysis, SNAPPE II scores were initially categorised as < 10 and ≥ 10 to demonstrate early separation of survival curves. This categorisation was used for illustrative purposes and not as a definitive prognostic threshold, as optimal SNAPPE II cut-offs vary across populations. Kaplan–Meier survival analysis (Fig. 2) stratified the 380 neonates into low-risk (< 10; n = 256) and high-risk (≥ 10; n = 124) groups.

Survival was significantly higher in the low-risk group (p < 0.0001, log-rank test). In the high-risk cohort, survival declined sharply within the first 40 days, whereas the low-risk group exhibited a more gradual decline, with a mortality plateau observed by day 90. At day 80, seven patients remained at risk in each group. These findings demonstrate clear survival discrimination between SNAPPE II risk groups in this surgical neonatal cohort.

**Fig. 3:**
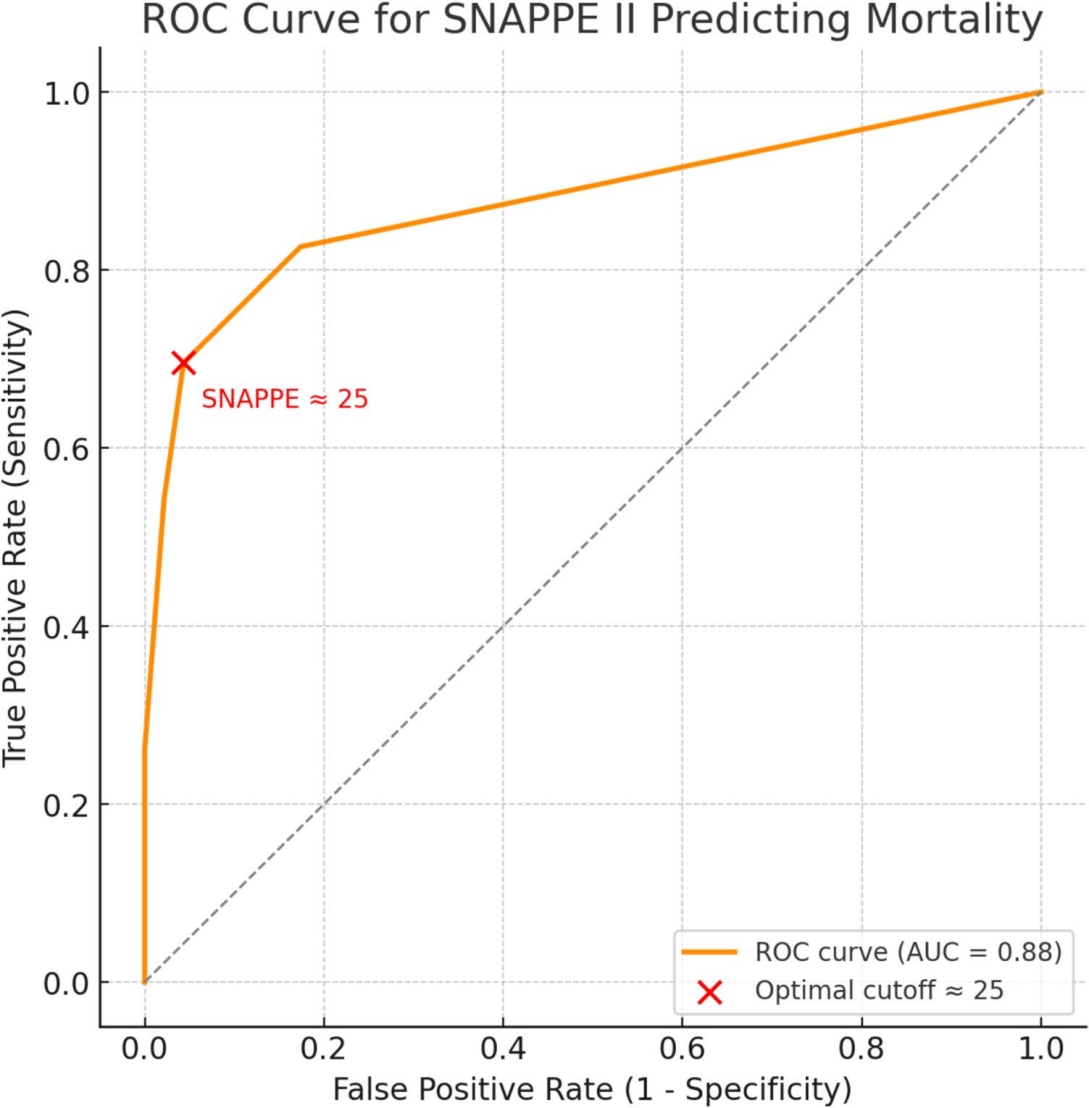
Receiver operating characteristic (ROC) curve showing the discriminative performance of SNAPPE II score across graded score ranges with the optimal cutoff annotated in red. The curve demonstrates excellent predictive ability with an area under the curve (AUC) of 0.88. Red marker shows optimal cutoff around SNAPPE II score 25 (Youden index maximum)

Receiver operating characteristic (ROC) analysis (Fig. 3) demonstrated excellent predictive performance of the SNAPPE II score for neonatal mortality, with an area under the curve (AUC) of 0.88. The optimal cut-off, defined by the maximum Youden index, occurred at a SNAPPE II score of 25, closely aligning with the observed clinical threshold of > 20, beyond which survival probability declined sharply.

Of the 124 neonates with SNAPPE II ≥ 10, 57.3% died; in contrast, only 5.9% died among the 256 with scores < 10. The score demonstrated a positive predictive value (PPV) of 57.3% and a negative predictive value (NPV) of 94.1%, indicating high reliability in identifying low-risk neonates. Sensitivity and specificity were 82.6% and 82.0%, respectively, with an overall accuracy of 82.1%. The phi coefficient (r = 0.576) denoted a moderate to strong association between SNAPPE II ≥ 10 and mortality.

## Discussion

Globally, approximately 1.7 billion children lack access to timely, safe, and affordable surgical care [9]. In Sub-Saharan Africa (SSA), where children comprise nearly 50.0% of the population, the region contributes disproportionately to global under-five mortality, much of it related to surgically correctable conditions [8, 10]. Neonates who die often present with multiple risk factors: prematurity, low birth weight, and surgical conditions linked to immaturity, such as necrotizing enterocolitis (NEC) [2, 15]. Outcomes in this cohort were strongly influenced by sepsis and the complex neonatal conditions prevalent in the local setting [7, 11, 16].

This study provides critical insight into the demographic and clinical characteristics of neonates admitted for surgical care at a quaternary referral centre. The population was predominantly male 209/380 (55.0%) and preterm 260/380 (68.0%), with a median age at admission of 4 days, reflecting early postnatal referral patterns common in tertiary neonatal units [17]. Notably, 239/380 (62.8%) of neonates had low birth weight, exceeding the global average of 14.6% reported by the World Health Organization [18]. The high prevalence of very low 50/380 (13.0%) and extremely low birth weight 7/380 (1.8%) underscores the severity of illness in this population, contributing to increased risks for NEC, sepsis, and intraventricular haemorrhage [19].

The gestational age distribution underscored the significant burden of prematurity, with 204/380 (54.0%) of neonates born moderate to late preterm and 57/380 (14.8%) born very or extremely preterm. A median gestational age of 36 weeks reflects this trend and highlights the critical need for enhanced antenatal surveillance and perinatal support systems. Furthermore, 118/380 (31.0%) of neonates were referred from distances exceeding 100 km. Longer referral distances may reflect systemic delays inherent to centralised surgical care in resource-limited settings [22]. While this study did not formally analyse distance-based outcome differences, the observed referral patterns highlight the geographic burden on tertiary paediatric surgical services.

Strikingly, only 9/380 (2.4%) of cases had a confirmed antenatal diagnosis, emphasizing gaps in prenatal screening and missed opportunities for early referral and multidisciplinary planning [23]. Timely identification of congenital anomalies could facilitate delivery at equipped centres and potentially mitigate morbidity and mortality.

This study also highlights diagnostic trends in surgical pathology. Complex gastroschisis was significantly associated with poor outcomes, consistent with literature emphasizing the risks of bowel necrosis, atresia, and volvulus in this subgroup [24]. These findings underscore the importance of stratifying disease severity within diagnostic categories to guide postoperative care and prognostication.

Postoperative morbidity occurred in 17.4% of cases, with surgical site infections (SSIs) being the most frequent complication (6.6%). This aligns with previous research emphasizing the vulnerability of neonates to infection due to immature immune function and prolonged exposure to invasive interventions [27].

The SNAPPE II score demonstrated strong predictive behaviour for mortality in this cohort. By quantifying physiological illness severity, SNAPPE II enables clinicians to anticipate risk, support prognostic discussions, and prioritise intensive care resources based on objective physiological derangement [28–30].

The observed mortality rate of 86/380 (22.6%) aligns with previously reported African rates ranging from 16.0 to 45.0% [4]. Sepsis emerged as the leading cause of death, responsible for 65.1% of mortalities. The predominance of Acinetobacter baumannii (32.1%), a multidrug-resistant organism, echoes global concerns about nosocomial infections in NICUs.

Culture-positive sepsis emerged as a critical determinant of mortality in this cohort. Neonates with positive blood cultures had a fivefold higher odds of death compared to those with negative cultures. This finding is consistent with prior studies demonstrating that bloodstream infections substantially worsen outcomes in surgical neonates [31, 32]. These data highlight the urgent need for timely sepsis recognition, empiric antimicrobial coverage, and strict infection control measures, particularly in high-dependency NICU environments where invasive procedures are common.

Diagnosis-specific mortality was highest in NEC 22/86 (25.6%) and gastroschisis 20/86 (23.3%), followed by intestinal and oesophageal atresia (12.8%), ARM (7.0%), CDH (7.0%) and 24.3% from other conditions. These data reflect the complexity of neonatal surgical conditions and the necessity for multidisciplinary care.

SNAPPE II demonstrated strong prognostic utility in this surgical neonatal cohort. Receiver operating characteristic analysis confirmed excellent discrimination (AUC = 0.88**)**. Mortality rose sharply with scores > 20, which signalled a critical inflection point, and no survivors were recorded above 40. Scores < 10 reliably identified neonates with favourable outcomes**,** supporting early reassurance and streamlined care.

The inflection point identified in our cohort (scores > 20 with an optimal cut-off near 25) is consistent with published NICU data. Several neonatal studies have demonstrated a marked increase in mortality at SNAPPE II scores > 20, although reported optimal cut-offs vary by population and case-mix. Samanta et al. identified > 20 as the optimal cut-off for mortality prediction in neonates with sepsis, while Mathur et al. reported a comparable threshold in mixed NICU populations [29, 30]. Our findings, therefore confirm the external validity of SNAPPE II in a high-burden African surgical cohort, reinforcing its global applicability for perioperative risk stratification.

This risk-based stratification enables timely clinical decision-making, targeted monitoring, and informed discussions with families. While lower SNAPPE II groupings were used for descriptive survival analysis, clinically meaningful prognostic discrimination in this cohort occurred at higher scores (> 20), with markedly increased mortality beyond this threshold. The simplicity and non-invasive nature of SNAPPE II, relying solely on routinely available clinical and laboratory parameters, make it particularly feasible for integration into neonatal surgical units, including settings with limited diagnostic resources. Importantly, risk can be assigned within hours of admission, allowing early triage, resource allocation, and prioritisation of intensive support where it is most needed.

With 380 surgical neonates, this study represents one of the largest single-centre African cohorts evaluating SNAPPE II in a surgical neonatal population. Although SNAPPE II was developed in mixed neonatal intensive care populations, our findings demonstrate that it retains prognostic relevance in a high-burden surgical setting. This extends existing evidence to an African quaternary referral context characterised by a high sepsis burden, complex surgical pathology, and referral-related delays. In such settings, early physiological risk stratification may support triage, prioritisation of intensive monitoring and organ support, and more objective counselling of families.

## Limitations

This study is limited by its retrospective, single-centre design. Long-term outcomes, including neurodevelopmental status and nutritional recovery after discharge, were not assessed. In addition, the low antenatal diagnosis rate limited evaluation of the impact of prenatal detection on outcomes, which may reflect both true underdiagnosis and incomplete documentation. Although SNAPPE II was not originally designed specifically for surgical neonates, its strong association with mortality in this cohort supports its use as a pragmatic marker of physiological illness severity in resource-limited surgical settings. The absence of multivariable modelling represents a limitation, and future prospective studies may explore adjusted analyses to further refine risk estimation in surgical neonatal populations.

## Conclusion

This study offers important insights into the surgical care of neonates at a high-volume centre in SSA. The cohort was predominantly premature, low birth weight, and presented with complex conditions. Sepsis was the principal cause of death. SNAPPE II proved a strong predictor of mortality. Complex gastroschisis and NEC were the most common fatal diagnoses. Outcomes achieved at NMCH are encouraging and comparable to other high-volume regional centres. Although still below the survival rates reported in high-income countries, these outcomes align with those observed at comparable neonatal surgical centres in sub-Saharan Africa, where resource constraints, high sepsis rates, and complex case mixes drive substantial variability in survival.

These findings support the adoption of routine SNAPPE II scoring upon NICU admission for surgical neonates, to guide clinical surveillance intensity, inform multidisciplinary planning, and enhance communication with families. Routine use of SNAPPE II may enable earlier escalation of care, improve prognostic transparency for families, and support more rational allocation of limited NICU resources. Further investment in neonatal surgical services, NICU infrastructure, and antenatal care systems is essential to improve outcomes in this vulnerable population.

## Supplementary Information

Below is the link to the electronic supplementary material.

Supplementary Figure 1: Geographic distribution map showing the referral origins of patients to NMCH. The NMCH is marked with a black dot in Johannesburg, South Africa, serving as the focal point. Figure 1 presents a geospatial distribution map of referring institutions (PDF 2855 KB)

Supplementary Figure 2: Distribution of SNAPPE II scores by outcome (PDF 149 KB)

Supplementary Figure 3: Morbidities classified by the Clavien-Dindo classification (PDF 124 KB)

Supplementary Figure 4: Post-operative complications encountered in the cohort (PDF 16 KB)

## Data Availability

Data underlying this study are available from the corresponding author on reasonable request.
