# Supplementary figures and images for: Outcomes of general paediatric surgical neonates managed at the Nelson Mandela Children’s Hospital, Johannesburg, South Africa
Source: Pediatr Surg Int. 2026 Feb 27;42(1):122. doi: 10.1007/s00383-026-06365-y (PMC12948784; doi:10.1007/s00383-026-06365-y)

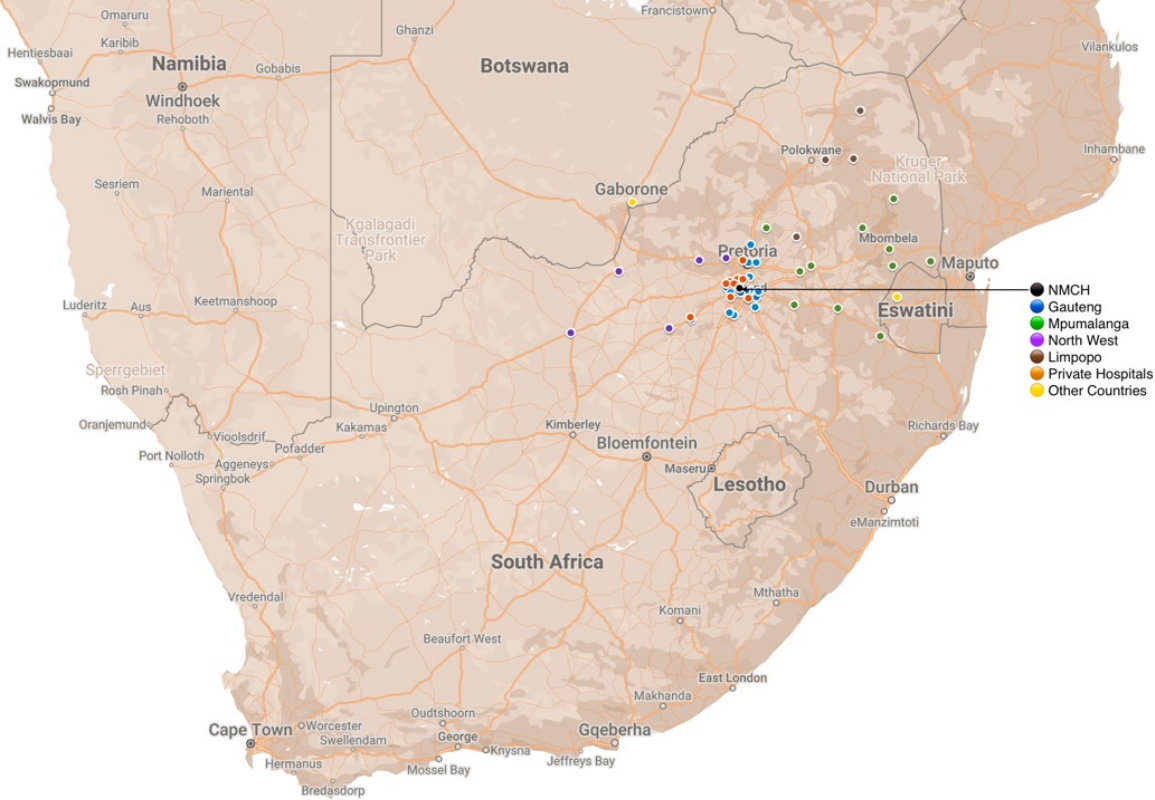

Supplement: Supplementary file 1 — Supplementary Figure 1: Geographic distribution map showing the referral origins of patients to NMCH. The NMCH is marked with a black dot in Johannesburg, South Africa, serving as the focal point. Figure 1 presents a geospatial distribution map of referring institutions (PDF 2855 KB) [file 383_2026_6365_MOESM1_ESM.pdf]

Distribution of SNAPPE II Scores by Outcome

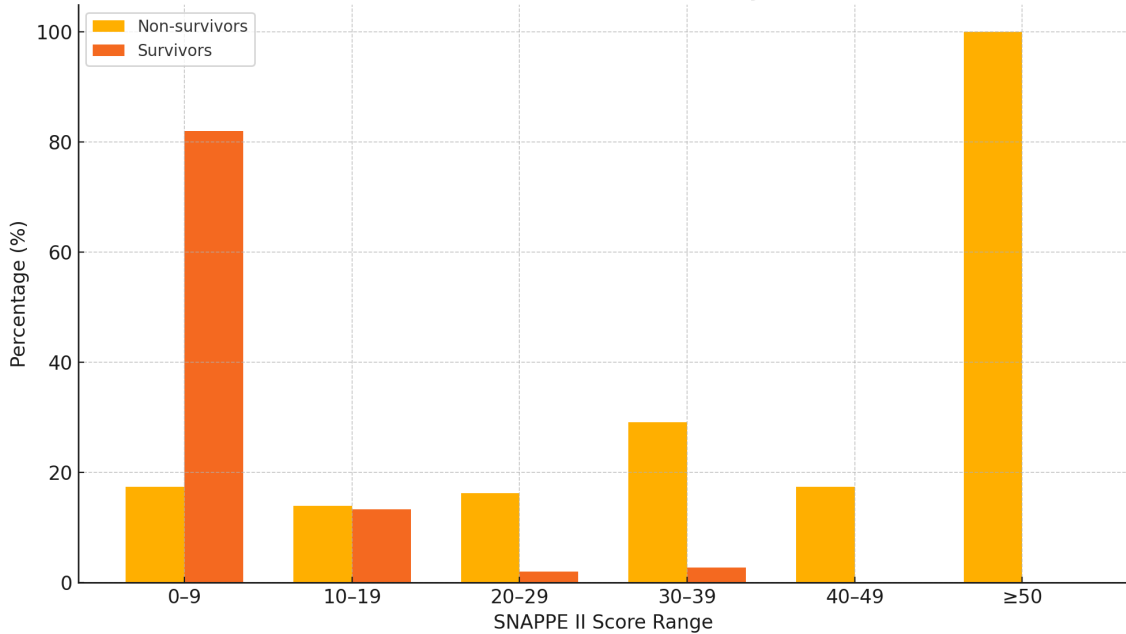

Supplement: Supplementary file 2 — Supplementary Figure 2: Distribution of SNAPPE II scores by outcome (PDF 149 KB) [file 383_2026_6365_MOESM2_ESM.pdf]

Morbidity (Clavien Dindo Classification)

Grade 5  
Grade 4b  
Grade 4a  
Grade 3b  
Grade 3a  
Grade 2  
Grade 1

0 5 10 15 20 25 30 35 40

Total

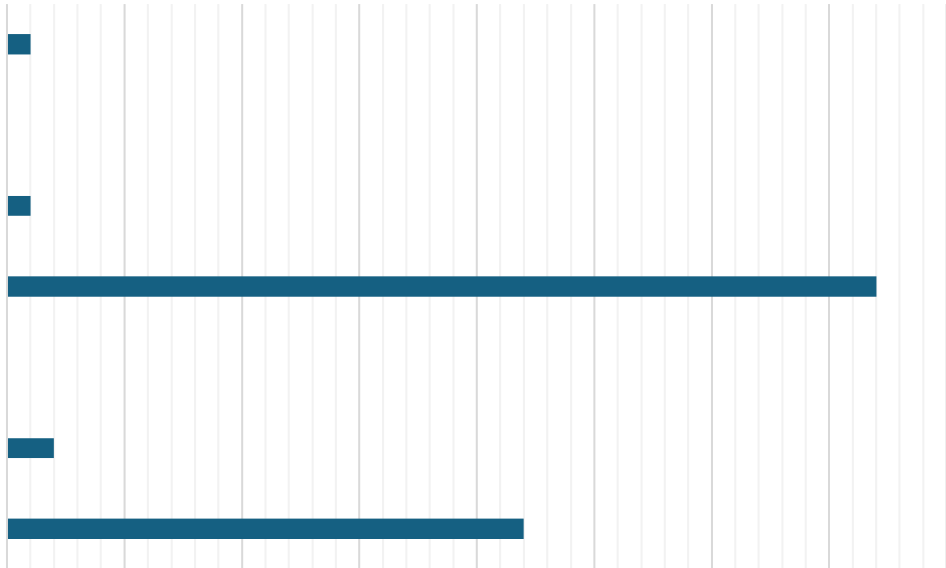

Supplement: Supplementary file 3 — Supplementary Figure 3: Morbidities classified by the Clavien-Dindo classification (PDF 124 KB) [file 383_2026_6365_MOESM3_ESM.pdf]

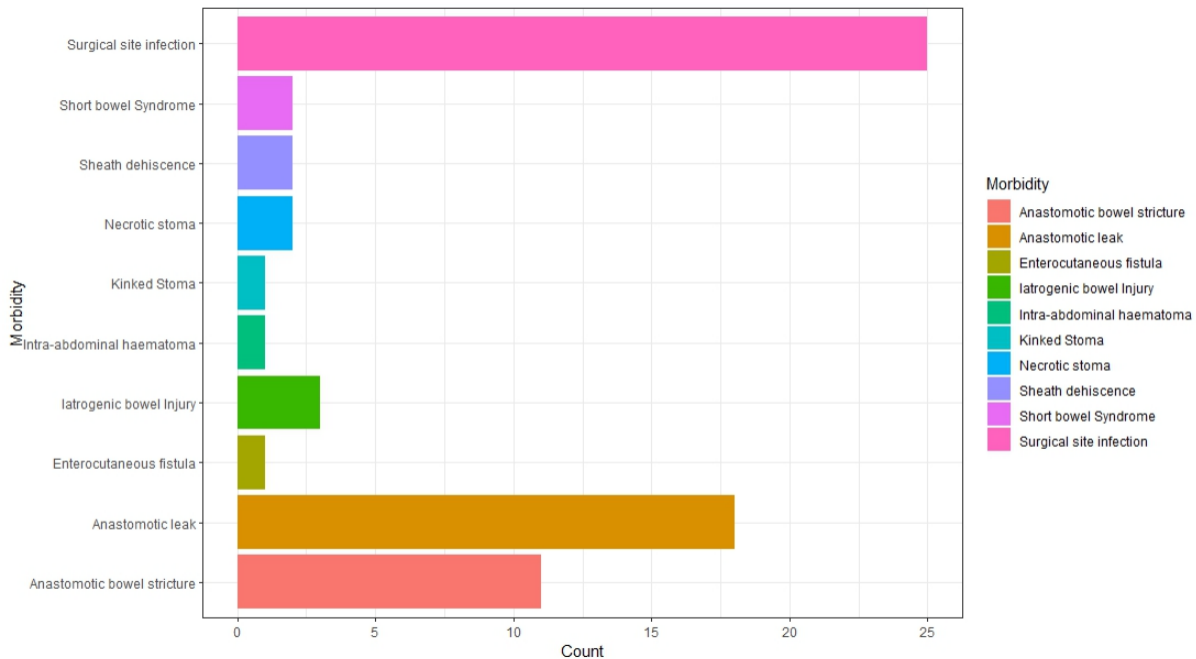

Supplement: Supplementary file 4 — Supplementary Figure 4: Post-operative complications encountered in the cohort (PDF 16 KB) [file 383_2026_6365_MOESM4_ESM.pdf]
